# Gillespie syndrome in a South Asian child: a case report with confirmation of a heterozygous mutation of the ITPR1 gene and review of the clinical and molecular features

**DOI:** 10.1186/s12887-018-1286-5

**Published:** 2018-09-24

**Authors:** Daham De Silva, Kathleen A. Williamson, Kavinda Chandimal Dayasiri, Nayani Suraweera, Vinushiya Quinters, Hiranya Abeysekara, Jithangi Wanigasinghe, Deepthi De Silva, Harendra De Silva

**Affiliations:** 1 Department of Paediatrics, Faculty of Medicine, University of Colombo, Colombo, 8 Sri Lanka; 2 MRC Human Genetics Unit, MRC Institute of Genetics and Molecular Medicine, University of Edinburgh, Edinburgh, UK; 3 Professorial Paediatric Unit, Lady Ridgeway Hospital for Children, Colombo, 08 Sri Lanka; 4 Department of Ophthalmology, Lady Ridgeway Hospital for Children, Colombo, 08 Sri Lanka; 5 Department of Physiology, Faculty of Medicine, University of Kelaniya, Kelaniya, Sri Lanka

**Keywords:** Gillespie syndrome, Partial aniridia, Cerebellar hypoplasia, ITPR1 gene

## Abstract

**Background:**

Gillespie syndrome is a rare, congenital, neurological disorder characterized by the association of partial bilateral aniridia, non-progressive cerebellar ataxia and intellectual disability. Homozygous and heterozygous pathogenic variants of the ITPR1 gene encoding an inositol 1, 4, 5- triphosphate- responsive calcium channel have been identified in 13 patients recently. There have been 22 cases reported in the literature by 2016, mostly from the western hemisphere with none reported from Sri Lanka.

**Case presentation:**

A 10-year-old girl born to healthy non-consanguineous parents with delayed development is described. She started walking unaided by 9 years with a significantly unsteady gait and her speech was similarly delayed. Physical examination revealed multiple cerebellar signs. Slit lamp examination of eyes revealed bilateral partial aniridia. Magnetic resonance imaging of brain at the age of 10 years revealed cerebellar (mainly vermian) hypoplasia. Genetic testing confirmed the clinical suspicion and demonstrated a heterozygous pathogenic variant c.7786_7788delAAG p.(Lys2596del) in the ITPR1 gene.

**Conclusion:**

The report of this child with molecular confirmation of Gillespie syndrome highlights the need for careful evaluation of ophthalmological and neurological features in patients that enables correct clinical diagnosis. The availability of genetic testing enables more accurate counseling of the parents and patients regarding recurrence risks to other family members.

## Background

Gillespie syndrome, also known as, aniridia-cerebellar ataxia-intellectual disability syndrome, is a rare form of congenital dysautonomia characterized by non-progressive cerebellar ataxia, partial aniridia, and intellectual impairment [1]. Since it was first reported in 1965 [2], there have been 22 cases been reported worldwide by 2016 [3, 4]. Most reports are consistent with an autosomal dominant or recessive pattern of inheritance [5]. Identification of homozygous or compound heterozygous recessive or de novo heterozygous dominant pathogenic variants restricted to particular domains of the inositol 1,4,5-trisphosphate receptor type 1 (ITPR1 gene) has confirmed its genetic cause [3, 4, 6]. We report the first South Asian Gillespie syndrome case with a heterozygous pathogenic variant [c.7786_7788 del AAG p. (Lys 2596del)] of the ITPR1 gene. This new case of Gillespie syndrome was found to carry the recurrent heterozygous ITPR1 pathogenic variant c.7786_7788delAAG p.(Lys2596del), which has been reported in seven other cases [3, 4, 6–8].

## Case presentation

The proband is a 10-year-old Sri Lankan girl of Sinhala ethnicity born to healthy non-consanguineous parents with two other older, healthy sons. She had an uneventful pregnancy and birth history and there was no family history of ophthalmological or neurological diseases. Although her parents noted delayed development, they had not sought medical attention for this. She started walking unaided at around 9 years and continues to have a significantly unsteady gait. Her hearing was not affected but speech was delayed (first word at 3 years and currently speaking 3–4-word complex sentences). Her parents reported photophobia since infancy.

**Fig. 1:**
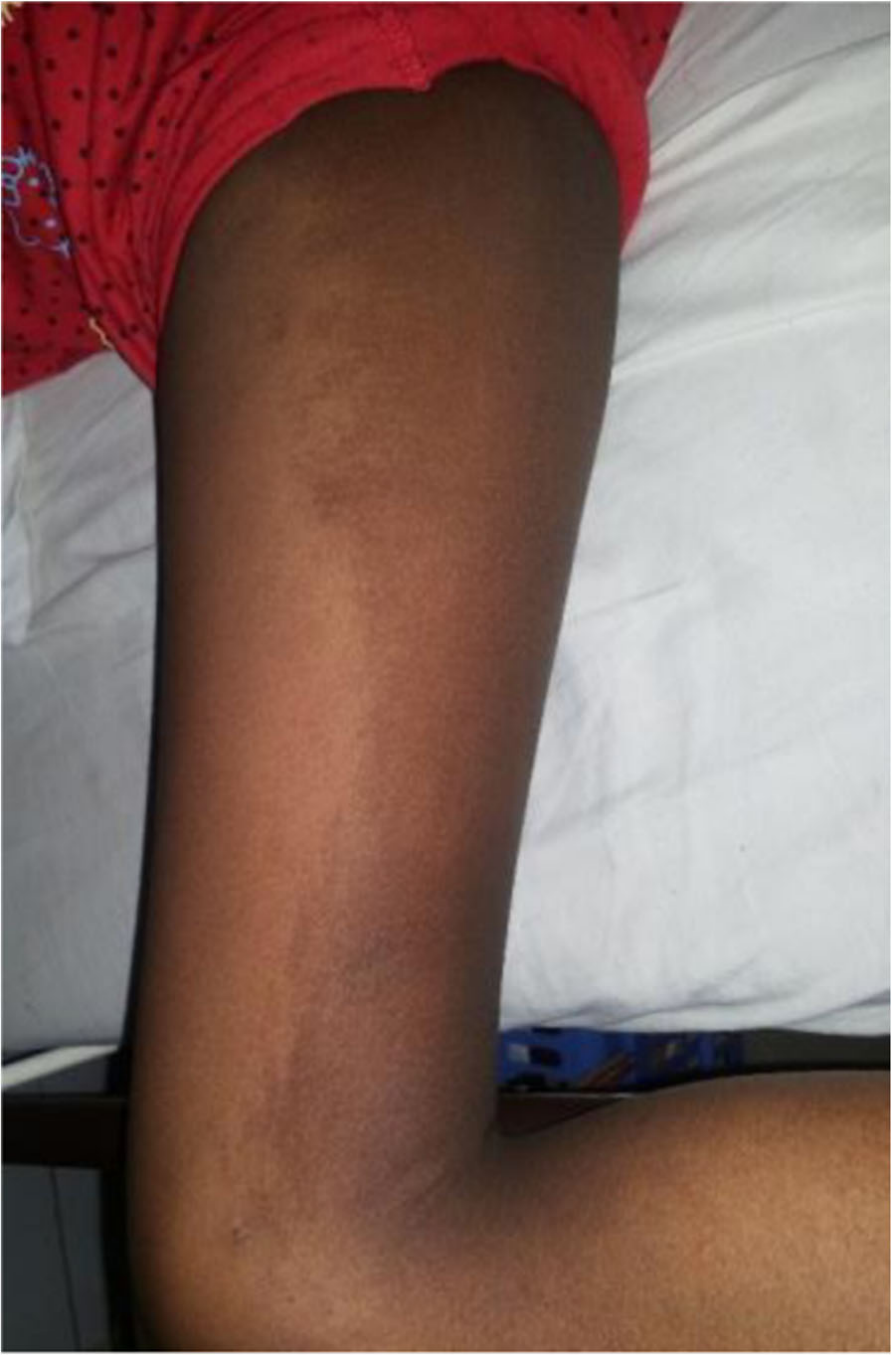
Right thigh with pigmented macule with a blaschcoid distribution

**Fig. 2:**
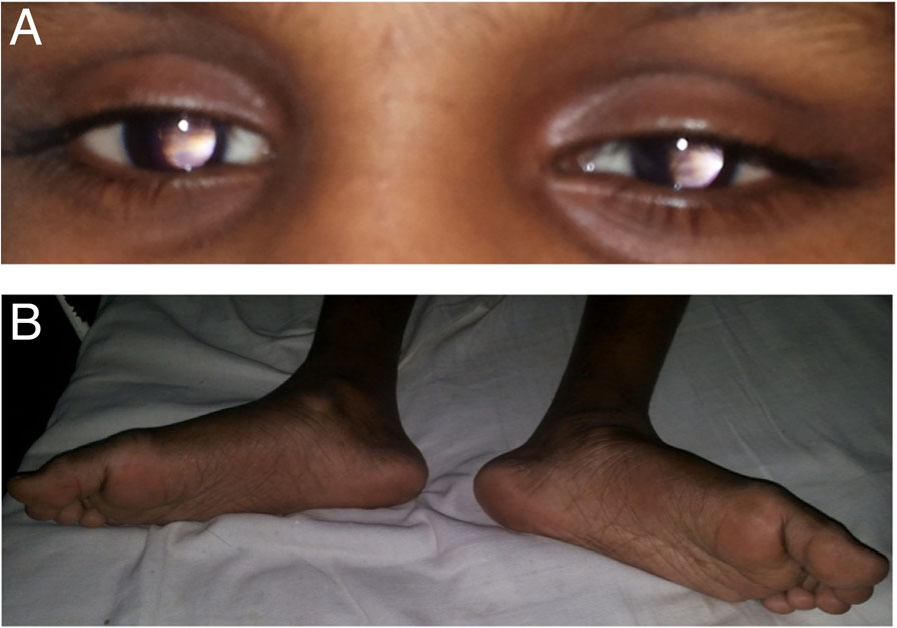
**a** Close up of eyes showing bilateral partial aniridia (**b**) Lower limbs with generalized wasting and pes planus

On examination aged 10 years, her height, weight and occipito-frontal circumference were 132 cm (10th–25th centile), 26 kg (25th–50th centile) and 51 cm (25th–50th centile) respectively. She had no dysmorphic features. A pigmented macule with a serpiginous border (22 cm × 10 cm), which may represent a blaschcoid pattern suggestive of a cutaneous mosaicism, was noted on her right thigh (Fig. 1). Neurological examination revealed an ataxic gait, hypotonia, dysdiadochokinesia, intention tremor, horizontal nystagmus and dysarthria, all consistent with cerebellar disease. No pyramidal or extrapyramidal signs were identified. She had bilateral pes planus (Fig. 2). Ophthalmological examination revealed her pupils to be fixed and dilated and slit lamp examination revealed bilateral partial aniridia (Fig. 2) with a scalloped edge of the irises, normal fundi and impaired pupillary light reflexes. Pupillary membrane remnants were not seen and the cornea and lens were clear. Visual acuity was reduced (bilateral 6/30). Intelligence Quotient (IQ) assessment revealed moderate learning disability with better verbal IQ (verbal comprehension, working memory and processing speed scales) than non-verbal IQ (Test of Nonverbal Intelligence – TONI version 3- score 70). Her short-term memory was within normal limits although she had deficits in long-term memory.

**Fig. 3:**
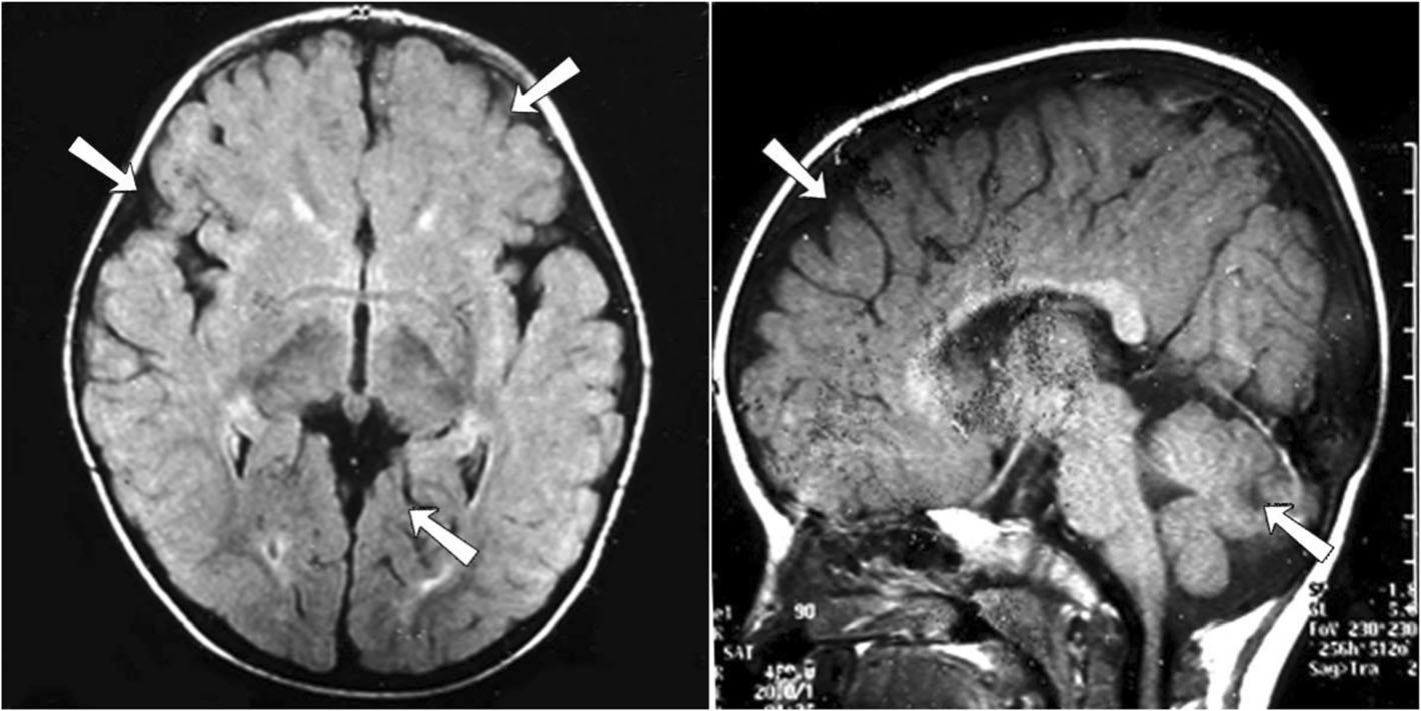
MRI brain performed at 10 years of age revealing cerebellar especially vermis hypoplasia, cerebral atrophy and white matter changes

Magnetic resonance imaging (MRI) of the brain revealed cerebellar hypoplasia especially affecting the vermis and white matter changes (Fig. 3). Cervical spine x-ray, echocardiogram and ultrasound abdomen were normal. Mutation analysis by region-of-interest targeted sequencing (NM_001168272.1/ENST00000302640 coding exons 46 and 52–56, which encode the region spanning Glu2094 and the entire calcium ion channel domain, respectively) was performed as previously described [4]. This identified a previously described pathogenic heterozygous variant in the ITPR1 gene, namely c.7786_7788delAAG p.(Lys2596del). The clinically unaffected parents’ DNA have not been tested for this variant due to lack of availability of samples.

## Discussion and conclusions

Gillespie syndrome was first described in two adult siblings who had the triad of partial aniridia, cerebellar ataxia and oligophrenia [2]. The syndrome has since then described in both males and females with variable ages of clinical diagnosis (1.5 years to 18 years) [3, 4].

This diagnosis needs to be considered in infants with aniridia associated with hypotonia. Bilateral partial aniridia is consistently seen in Gillespie syndrome. It has a characteristic scalloped pupillary border, iris strands with regularly spaced attachments to the anterior lens with some reports of persistence of the pupillary membrane. Complete aniridia, especially when associated with foveal hypoplasia, cataracts, corneal opacification with only rare extraocular involvement is usually inherited as an autosomal dominant trait; mutations of the Paired box protein 6 (PAX6) gene are commonly implicated [9]. Contiguous gene deletions involving PAX6 and associated with Wilms tumor, genital abnormalities and developmental delay is recognized as the WAGR syndrome. The PAX6 gene was studied for its possible association with Gillespie syndrome and found to be not associated in several reports [10–12]. Other genes are also associated with aniridia including the forkhead box C1 (FOXC1) and Paired-like homeobox transcription factor 2 (PITX2) but these are also associated with other, especially anterior chamber anomalies.

The second major diagnostic criterion for Gillespie syndrome is the involvement of the brain and in particular, the cerebellum. In early life, the majority of cases appear to have hypotonia with marked delay in gross and fine motor milestones being commonly reported in later infancy and childhood [3]. Cerebellar signs reflect primary cerebellar dysfunction and secondary disruption of the cerebro-cerebellar anatomical circuitry. The brain MRI scan of this child at 10-years of age revealed cerebellar atrophy and the neurological disability was similar to most previously reported cases [5]. There are reports of the progression of the cerebellar atrophy with age although the ataxia is usually reported to be non-progressive. In some cases, there are reports of additional cerebral white matter abnormalities suggesting the likelihood of more extensive neurological impairment [13]. Psychometric assessment in this patient confirmed moderate learning disability with better verbal IQ as compared to performance IQ. Similar findings in intellectual assessment have been reported in a recently published report of Gillespie syndrome [5].

Facial dysmorphism [14], pectoral agenesis and kyphosis are other reported manifestations [3, 4]. Bilateral pes planus deformity was not described in previously reported children.

The genetic basis of this syndrome was defined with identification of pathogenic variants in the inositol 1,4,5-trisphosphate receptor type 1 (ITPR1) gene on chromosome 3p26 [3, 4]. Both biallelic homozygous or compound heterozygous and usually de novo monoallelic heterozygous *ITPR1* gene pathogenic variants are the underlying genetic defects for autosomal recessive and dominant Gillespie syndrome respectively. The *ITPR1* gene encodes one of the three subtypes of the inositol triphosphate (IP_3_)-receptor family that form Ca^2+^ release channels especially in the endoplasmic reticulum. It is also expressed in the nervous system especially the Purkinje cells of the cerebellum [15]. The recessive pathogenic variants are predicted to cause loss of function of the ITPR1 protein while the dominant pathogenic variants appear to impair its function by interacting with the normal protein complex (dominant negative effect). The pathogenic variants are restricted to several residues of ITPR1 in the transmembrane region of the protein involved in forming a calcium transport channel. Genetic testing revealed a heterozygous, three base pair, in frame deletion in the ITPR1 gene namely ITPR1 c.7786_7788delAAG p. (Lys2596del) in this child. This pathogenic variant has been previously described in affected cases. As parental samples were not investigated, it is uncertain if this pathogenic variant is de novo or inherited from an unaffected parent. The ITPR1 gene has been implicated in spinocerebellar ataxia 15 (SCA 15), an autosomal dominant, slowly progressive ataxia with cerebellar atrophy but no aniridia. In SCA15, a milder, later onset form of cerebella ataxia, the commonest pathogenic variant is a heterozygous deletion. Early onset, non-progressive cerebellar ataxia (SCA29), infantile onset spinocerebellar ataxia and ataxic cerebral palsy have also been reported with missense pathogenic variants of ITPR1 but they differ from those in Gillespie syndrome [4].

Clinical diagnosis of Gillespie syndrome can be made following identification of its two cardinal features of cerebellar ataxia and partial aniridia. All cases of aniridia require careful clinical evaluation for additional ocular and extra-ocular features. Once suspected, targeted genetic testing will confirm the diagnosis and enable counseling regarding prognosis and risk of the disease in other family members. As the parents were not planning more family, the confirmation of a de novo pathogenic variant or inheritance from an unaffected parent was not pursued. This may be required if her clinically unaffected brothers wish to ascertain their status once they are older. Although this child’s testing was performed free of charge, the costs of genetic testing remain a barrier for accurate genetic diagnosis in patients from developing countries.

## Abbreviations

FOXC1 gene: Forkhead box C1 gene
IP_3_: Inositol triphosphate
IQ: Intelligence quotient
ITPR1 gene: Inositol 1,4,5-trisphosphate receptor type 1 gene
MRI: Magnetic resonance imaging
Pax 6: Paired box protein 6
PITX2: Paired-like homeobox transcription factor 2
SCA: Spinocerebellar ataxia
TONI: Test of nonverbal intelligence
WAGR syndrome: Wilms tumour, aniridia, genitourinary anomalies and mental retardation syndrome
